# Recent strengthening of the stratospheric Arctic vortex response to warming in the central North Pacific

**DOI:** 10.1038/s41467-018-04138-3

**Published:** 2018-04-27

**Authors:** Dingzhu Hu, Zhaoyong Guan, Wenshou Tian, Rongcai Ren

**Affiliations:** 1 Key Laboratory of Meteorological Disasters of China Ministry of Education (KLME), Joint International Research Laboratory of Climate and Environment Change (ILCEC), Collaborative Innovation Center on Forecast and Evaluation of Meteorological Disasters (CIC-FEMD), Nanjing University of Information Science & Technology, 210044 Nanjing, China; 2 College of Atmospheric Sciences, Lanzhou University, 730000 Lanzhou, China; 3 State Key Laboratory of Numerical Modeling for Atmospheric Sciences and Geophysical Fluid Dynamics, Institute of Atmospheric Physics, Chinese Academy of Sciences, 100029 Beijing, China

## Abstract

The stratospheric Arctic vortex (SAV) plays a critical role in forecasting cold winters in northern mid-latitudes. Its influence on the tropospheric mid- and high-latitudes has attracted growing attention in recent years. However, the trend in the SAV during the recent two decades is still unknown. Here, using three reanalysis datasets, we found that the SAV intensity during 1998–2016 has a strengthening trend, in contrast to the weakening trend before that period. Approximately 25% of this strengthening is contributed by the warming of sea-surface temperature (SST) over the central North Pacific (CNP). Observational analysis and model experiments show that the warmed CNP SST tends to weaken the Aleutian low, subsequently weakening the upward propagation of wavenumber-1 planetary wave flux, further strengthening the SAV. This strengthened SAV suggests important implications in understanding the Arctic warming amplification and in predicting the surface temperature changes over the northern continents.

## Abstract (web summary)

The stratospheric Arctic vortex plays a critical role in forecasting cold winters in northern mid-latitudes. Here the authors show that the stratospheric Arctic vortex strengthened during 1998–2016, with ~25% of this strengthening contributed by warming in the central North Pacific.

## Introduction

The stratospheric Arctic vortex (SAV) and its evolution play a critical role in stratosphere–troposphere coupling^1^, and the weather and climate variations at northern mid- and high-latitudes^2–4^. The anomalously warm SAV may cause severely cold winters and frequent cold air outbreaks at northern mid-latitudes^4–7^. Because of its significant influence on the tropospheric weather and the climate, a growing number of studies have focused on the variability of the SAV^4,8^. Some studies have found that the SAV was much cooler during 1950–2000^9^. However, the temperature in the lower Arctic stratosphere has exhibited a warming trend since 1990, based on ERA-Interim (European Centre for Medium-Range Weather Forecasts interim reanalysis), MERRA (Modern-Era Retrospective Analysis for Research and Applications), and JRA-55 (Japanese 55-year Reanalysis)^10^. This trend can also be detected in ERA-40 (40-year European Centre for Medium-Range Weather Forecasts Re-Analysis)^11^, although ERA-40 is poorly suited to studying the vertical profile and trends of Arctic temperature^12^. This warming trend in the Arctic stratosphere is consistent with the increasing of the stratospheric wave flux under global warming conditions^10,13^, implying a weakening of the SAV since the 1990s. However, many recent studies have reported that various aspects of the climate indicate a turning point around 2000s^14–18^. For example, the warming trend of global mean-surface temperature slowed down during 1998–2012^14,15,19^. Some studies have attributed this recent warming to the internal climate variability^14,15,19–21^. An interesting question, however, is whether the SAV also experienced a turning point around 2000s.

Sea-surface temperature (SST) is thought to be an important cause of SAV variability^22,23^, with warmer SSTs tending to weaken the SAV by changing the planetary wave propagation from the troposphere into the stratosphere^22^. However, SST changes over different regions have different impacts on the SAV at different timescales. Some studies have argued that SST changes over tropical oceans—particularly the SST changes associated with El Niño–Southern Oscillation (ENSO) events—have significant impacts on SAV variability^24,25^. In addition, weakening of the SAV during winter is closely connected with cooling of North Pacific^23,26^. A recent study revealed that the North Pacific exhibited a greater warming trend during 1999–2012^15^. Based on this warming trend in the North Pacific after 2000s, we examined whether the SAV strength responds actively to warming of the North Pacific during the recent two decades.

In this study, we use several reanalysis datasets and a chemistry-climate model to investigate the trend in the SAV strength in boreal winter during 1998–2016, and its connections to the North Pacific. Our results show that the SAV strengthens during this period, one-quarter of which can be associated with warming of the North Pacific through reducing the upward wavenumber-1-planetary wave flux in the extratropical stratosphere. A stronger SAV implies that the weather and the climate in the northern mid- and high-latitudes are likely to change markedly in the future. Our results are essential for understanding the stratosphere–troposphere coupling, and may provide clues to improve the winter-time forecasts of high-impact weather events at mid-latitudes in the Northern Hemisphere.

## Results

### Strengthening of the SAV

**Fig. 1:**
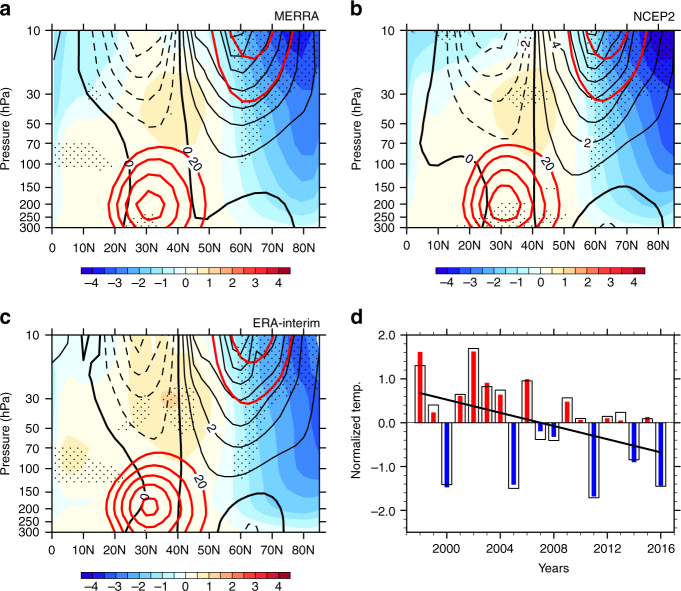
Temperature and zonal winds trends in the Arctic stratosphere. Temperature (shading, units: K decade^−1^) and zonal wind (black contours; interval 1 m s^−1^ decade^−1^) trends during 1997/98–2015/16 for December–January–February derived from MERRA (**a**), NCEP2 (**b**), and ERA-Interim (**c**). Climatological zonal winds >20 m s^−1^ are shown with thick red contours at a contour interval of 5 m s^−1^. Areas where the temperature trends are significant at/above the 95% level are stippled. The normalized time series of stratospheric temperature averaged over 65°N–80°N from 50 up to 10 hPa from MERRA (broad open bars) and NCEP2 (narrow filled bars) are shown in **d**. The straight black line indicates the linear trend in the normalized stratospheric polar temperature using MERRA

The December, January, and February (DJF) mean temperature over the Arctic from 1997/98 to 2015/16 has a cooling trend in the stratosphere with the largest cooling in the mid-stratosphere (Fig. 1). This statistically significant cooling trend can be detected in the temperature fields from three reanalysis datasets [MERRA, NCEP2 (National Centers for Environmental Prediction–Department of Energy Global Reanalysis 2), and ERA-Interim] from 1997/98 to 2015/16. Although some studies have shown that certain discrepancies still exist between different reanalysis datasets^27^, the strengthening trend in the SAV during 1998–2014 is evident in all the different reanalysis datasets (Fig. 1). The time series of temperature averaged over (65°N–90°N, 50–10 hPa) during 1997/98–2015/16 (Fig. 1d) also shows a statistically significant (above 95% confidence level) cooling trend in all the three reanalysis datasets, with a linear trend of –0.75 ± 0.67 K decade^−1^ from MERRA, –0.79 ± 0.67 K decade^−1^ from NCEP2, and –0.73 ± 0.68 K decade^−1^ from ERA-Interim.

Consistent with the thermal-wind balance, the westerly winds over the Arctic stratosphere during 1997/98–2015/16 show a strengthening trend. These trends in temperature and westerly winds in the Arctic stratosphere above 300 hPa during the past two decades indicate that the SAV has been strengthening, in contrast to the weakening of the SAV during 1990–2012^10^ and 1979–2008^11^. The warming trend in the Arctic stratospheric temperature from 1979 to around 2000 is indeed visible in several reanalysis datasets (Supplementary Fig. 1) and previous studies has attributed this trend to the enhanced upward propagation of the planetary waves in the extratropical troposphere^13,22,24^. However, a cooling trend in the Arctic stratosphere during 1997/98–2015/16 is evident in different reanalysis datasets (Supplementary Fig. 1), and this trend keeps to be robust when the start point of the time series is shifted several years earlier or later (Supplementary Figs. 2, 3). Note that during 1998–2016, the trends in the Arctic temperature showed similar patterns in the lower- and mid-stratosphere, displaying that the Arctic cooling (Supplementary Fig. 4) prevailed in the lower- and mid-stratosphere during 1998–2016.

### Dynamical processes

**Fig. 2:**
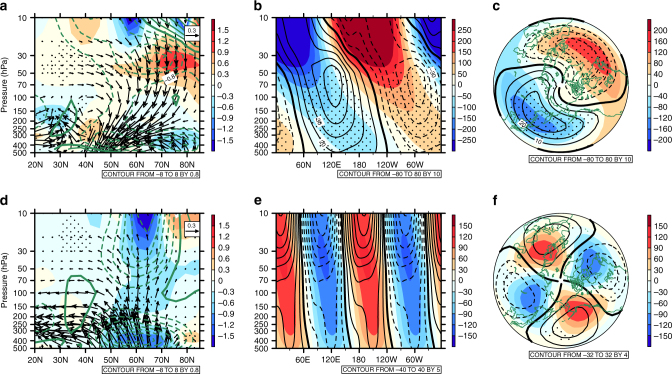
Trends in the wave flux. The trends in the Eliassen–Palm (EP) flux vectors (arrows with units of 10^4^ kg s^−2^ decade^−1^ for vertical vectors, and 10^6^ kg s^−2^ decade^−1^ for horizontal vectors) and the EP flux divergence (shading) (**a**, **d**), the geopotential height (contours; units: gpm decade^−1^) averaged over 45°N–75°N (**b**, **e**), and geopotential height (contours) at 200 hPa (**c**, **f**) with wavenumber-1 wave (**a**–**c**) and wavenumber-2 wave (**d**–**f**) during 1997/98–2015/16 for December–January–February using MERRA. The green solid and dashed contours in **a** and **d** indicate the positive and negative divergences of the climatological EP flux, respectively, whereas the stippled areas in these two panels show the trends significant at/above the 95% confidence level. Shaded contours in **b**, **e**, **c**, **f** indicate the mean climatology of the geopotential heights

To understand why the SAV strengthens, we examine the wave activity in the stratosphere by using the Eliassen–Palm (EP) flux^26,28^. The tropospheric waves that can propagate into the stratosphere are the predominant wavenumber-1 and wavenumber-2 waves^29^. Figure 2a, d shows the trends in the wavenumber-1 and wavenumber-2 components of the EP fluxes and their divergences, respectively, during 1997/98–2015/16 using MERRA. The upward propagation of the wavenumber-1 wave from the troposphere into the stratosphere weakens and its EP flux convergence over the mid- and high-latitudes decreases during 1997/98–2015/16 (Fig. 2a). The suppressed EP fluxes associated with the wavenumber-1 wave converge in the extratropical lower- and mid-stratosphere, which co-occurs with the weakening of the upward propagation of the wavenumber-1 planetary wave from the upper troposphere into the stratosphere (Fig. 2a). However, the EP fluxes associated with the wavenumber-2 wave (Fig. 2d) increase in the mid-latitude stratosphere, and the EP flux convergence has an increasing trend in the mid-latitude troposphere and in the mid- and high-latitude lower stratosphere.

The decreased upward EP fluxes of the wavenumber-1 planetary wave and the corresponding EP flux divergence changes in the Northern Hemisphere extratropical stratosphere (Fig. 2a) coincide with the decrease in temperature and the strengthening of the westerly winds in the extratropical lower- and mid-stratosphere (Fig. 1). Conversely, the EP fluxes associated with the wavenumer-2 planetary wave have opposite trends to that of the wavenumber-1 planetary waves. The weakening (strengthening) upward propagation of the wavenumber-1 (wavenumber-2) planetary wave into the stratosphere can also be observed in the three reanalysis datasets (Supplementary Fig. 5), suggesting that the strengthening of the SAV (Fig. 1) is indeed closely related to the weakening of the upward propagation of the wavenumber-1 planetary wave.

The longitudinal and vertical structure of the geopotential height averaged over 45°N–75°N (Fig. 2b, e) shows that the regions with negative (positive) trends of the zonal wavenumber-1 component of geopotential height anomalies are collocated with regions with positive (negative) climatological geopotential height anomalies. However, trends in the zonal wavenumber-2 component of geopotential height anomalies are opposite to those of the wavenumber-1 component of geopotential height anomalies. In boreal winter during 1997/98–2015/16, trends in geopotential height anomalies associated with wavenumber-1 (wavenumber-2) planetary waves at 200 hPa are mostly out-of-phase (in-phase), with their climatologies in the upper troposphere (Fig. 2c, f). This suggests that the wavenumber-1 (wavenumber-2) planetary wave in the upper troposphere has weakened (intensified) during the past two decades. Therefore, the decreased upward planetary wave flux in the stratosphere is mainly due to the weakening of the wavenumber-1 planetary wave in the upper troposphere, while the strengthening of the wavenumber-2 planetary wave acts to partly offset the strengthening of the SAV caused by the weakening of wavenumber-1 planetary waves.

**Fig. 3:**
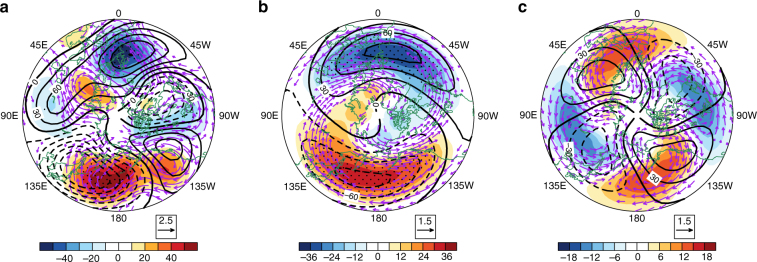
Trends in the wave activity. The trends in the geopotential height (shading; units: gpm decade^−1^) and winds (vectors; m s^−1^ decade^−1^; only values above 1.0 m s^−1^ decade^−1^ are shown) at 500 hPa using MERRA during December–January–February over 1997/98–2015/16 (**a**). **b**, **c** As in **a**, but for **b** wavenumber-1 and **c** wavenumber-2 components. The black contours indicate the mean climatology of the 500-hPa geopotential height. The stippled areas show the trends significant at/above the 95% confidence level

To further trace the wave activity changes during the past two decades, Fig. 3a–c shows the trends in the geopotential height and zonal winds at 500 hPa and their wavenumber-1 and wavenumber-2 components for DJF during 1997/98–2015/16. A statistically significant increasing trend in the 500 hPa geopotential height over the North Pacific, accompanied by an anticyclonic circulation trend, is evident during the past two decades (Fig. 3a). Interestingly, similar trends also occur in corresponding components of the wavenumber-1 (Fig. 3b) and wavenumber-2 (Fig. 3c) geopotential height and zonal wind anomalies over the North Pacific. The above results suggest that the Aleutian low has experienced a weakening trend during the past two decades. The positive west Pacific (WP) pattern and the negative Pacific-North America (PNA) pattern are both characterized by a weak Aleutian low^30^. Trends in WP and PNA indices, based on the definition by Wallace and Gutzler^30^, for the past two decades, are 0.38 and −0.23 decade^−1^, respectively, albeit they are statistically insignificant. Previous studies have shown that a stronger WP pattern tends to inhibit the driving of the planetary waves, which further strengthens the Arctic vortex^22,25,31,32^. Other studies have concluded that a weaker PNA pattern is associated with a weakened-planetary wavenumber-1 wave in the extratropical stratosphere^33^. Here, the positive trend of the WP index and the negative trend of the PNA index during 1998–2016 mainly result from the increased geopotential height at 500 hPa over the North Pacific, which has the largest and most significant trend (Fig. 3). The weakened Aleutian low tends to cancel (reinforce) the climatological wavenumber-1 (wavenumber-2) planetary wave, which causes a weakening (strengthening) of the wavenumber-1 (wavenumber-2) planetary wave (Fig. 3b, c). The anomalous wavenumber-1 and wavenumber-2 components of the eddy heat flux can be estimated into individual contributions from the interference and the wave-packet terms, according to Nishii et al.^34,35^. Our analysis indicates that the weakened (strengthened) upward propagation of the planetary wavenumber-1 (wavenumber-2) wave in the stratosphere is dominated by the interference between climatological planetary waves and associated anomalies (Supplementary Fig. 6). The weakened Aleutian low along with the lowered critical layer are mainly responsible for the decreasing trend in the upward wave flux from the upper troposphere into the extratropical stratosphere (Fig. 2a).

The polar cap height (PCH) anomaly is another good indicator of the variability of the polar vortex^36^. Here, the sign-reversed geopotential height (NPCH) anomalies averaged over 65°N–90°N at 50 hPa are used to describe the strength of the SAV, i.e., a positive (negative) NPCH anomaly corresponds to a strengthened (weakened) SAV. The geopotential heights, winds, and wavenumber-1 and wavenumber-2 components of the EP fluxes along with their divergences are regressed onto the normalized DJF mean NPCH for the period 1997/98–2015/16 (Supplementary Fig. 7). The spatial patterns of these regressed quantities suggest that a strengthened SAV corresponds to a weakened Aleutian low and a weakened-wavenumber-1 planetary wave propagation into the stratosphere, which are consistent with the weakening trend of the Aleutian low (Fig. 3a) and the wavenumber-1 planetary wave (Fig. 2a) during the past two decades. These further confirm that the strengthening trend of the SAV is closely related to the weakened-wavenumber-1 planetary wave induced by a weakened Aleutian low.

### Central-North-Pacific-Arctic vortex relationship

**Fig. 4:**
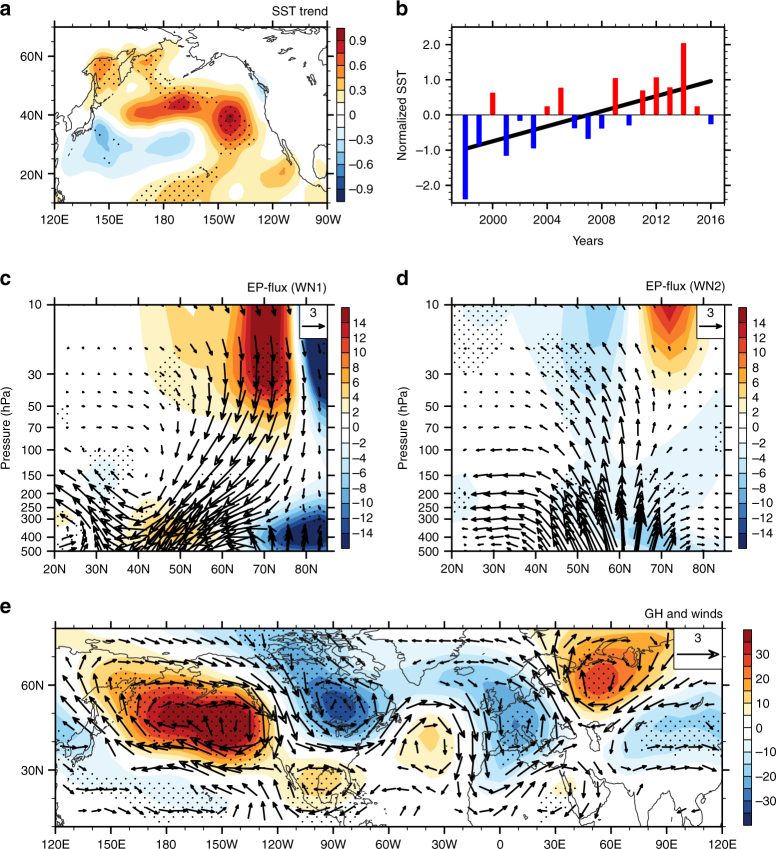
Wave activity linked to the central North Pacific SST. Trends in the North Pacific SST (shading; units: K decade^−1^) during boreal winter using the ERSST.v3b dataset from 1997/98 to 2015/16 (**a**). Time series of the normalized central North Pacific SST averaged over (35°N–45°N, 150°E–150°W; SST_CNP_) (**b**). The straight black line represents the linear trend in the normalized time series of SST_CNP_. The Eliassen–Palm (EP) flux and its divergence with **c** wavenumber-1 and **d** wavenumber-2 components from MERRA regressed onto the normalized SST_CNP_ in DJF during 1997/98–2015/16, respectively. **e** As in **c** and** d**, but for regressions of 500-hPa geopotential height anomalies (contours; units: 100 gpm) and winds (vectors; only values >0.5 m s^−1^ are shown). Geopotential height trends in stippled areas are significant at/above the 95% confidence level

It is not clear what leads to weakening of the Aleutian low. It has been shown that the higher SSTs in the North Pacific, which result in an intensified WP teleconnection pattern, are accompanied by a weaker Aleutian low and a more stable and persistent Arctic vortex^22^. Figure 4a shows the SST trends over the North Pacific derived from the ERSST.v3b (Extended Reconstructed SST, version 3b) dataset from 1997/98 to 2015/16. SSTs exhibit significant positive trends over the central North Pacific (CNP) and non-significant negative trends over the subtropical WP and areas along the west coast of North America and Alaska (Fig. 4a). To clarify the relationship between the CNP SST and the SAV, a CNP SST index (SST_CNP_) has been defined as SST anomalies averaged over the region (35°N–45°N, 150°E–150°W), where SST trends are statistically significant. The correlation coefficient between the normalized SST_CNP_ and the Pacific Decadal Oscillation (PDO) index is –0.64 during 1900–2016, which means that SST_CNP_ variations are closely related to the PDO. This is also supported by the fact that a negative phase of the PDO began in 1999, and a warming trend could be identified in the North Pacific SST during 1999–2012^15^.

Time series of the normalized SST_CNP_ is shown in Fig. 4b. Fluctuations in the SST_CNP_ are out-of-phase with variations in the temperature in the Arctic stratosphere (Fig. 1d). The correlation coefficient between the original (detrended) SST_CNP_ and the temperature in the Arctic stratosphere averaged over 65°N–90°N and 50–10 hPa during 1997/98–2015/16 is −0.40 (−0.23) in MERRA, −0.48 (−0.33) in NCEP2, and −0.42 (−0.28) in ERA-Interim, respectively. Furthermore, spatial patterns of the 50 hPa geopotential height and its wavenumber-1 and wavenumber-2 components regressed onto normalized time series of the DJF mean SST_CNP_ (Supplementary Figs. 8d–f) are overall similar to their corresponding trends (Supplementary Figs. 8a–c). This suggests that the recent strengthening of the SAV is related to the warming of CNP SST during the past two decades.

The pattern of regression coefficients of the geopotential height and winds on the normalized SST_CNP_ exhibits a statistically significant increasing trend in the geopotential height along with an anticyclonic anomaly in winds over the North Pacific (Fig. 4e). These resemble the trends of the geopotential height and winds at 500 hPa (Fig. 3a), and are also similar to those regressed onto the normalized NPCH index (Supplementary Fig. 7). The normalized SST_CNP_ is also closely correlated with the WP (*r* = 0.55) and PNA (*r* = −0.61) indices at 500 hPa over 1998–2016, indicating that the SST increases over the CNP correspond to a weaker Aleutian low. The EP fluxes and the EP flux divergence associated with the wavenumber-1 and the wavenumber-2, derived from the regressed disturbances on the normalized SST over the CNP (Fig. 4c, d), display similar variations to those shown in Fig. 2a, d. It means that there is a weakening in the upward wave flux associated with the wavenumber-1 planetary wave and an increase in the upward wave flux associated with the wavenumber-2 planetary wave from the upper troposphere into the extratropical stratosphere when the CNP SST becomes warmer.

It is known that stationary wave energy propagation plays an important role in connections between the SST and the Arctic climate^37–39^. The regression map of the geopotential height and streamfunction anomalies at 200 hPa with respect to the normalized CNP SST index clearly exhibits a Rossby wave train at mid-latitudes with alternating negative and positive geopotential height anomalies extending from the North Pacific into the Eurasian continent, along with alternating anticyclonic and cyclonic anomalies in the 200 hPa winds (Supplementary Fig. 9a, b). Previous studies have showed that the tropospheric stationary wave train at mid-latitudes is closely related to the Rossby wave source (RWS) associated with SST anomalies^40^. We further calculated RWS^41^ anomalies associated with the CNP SST and found negative anomalies of the RWS over the subtropical North Pacific in response to the warmer CNP SSTs (Supplementary Fig. 9c), which are accompanied by an anomalous divergent flow at 200 hPa. These indicate that RWS anomalies may originate from the anomalous upper level divergence associated with the warmer CNP SST, which is consistent with an ascending motion in response to the CNP warming there (Supplementary Fig. 10). Meanwhile, the geopotential height over high-latitudes in response to the warmed CNP exhibits negative anomalies, which are accompanied with downward motions there. As the westerly jet acts as a waveguide for Rossby wave propagations^42^, a significant weakened jet stream over the North Pacific in the troposphere is associated with the warmed CNP SST (Supplementary Fig. 9d), which corresponds to a weakened Aleutian low (Supplementary Fig. 9a) and an anticyclonic streamfunction anomalies (Supplementary Fig. 9b) over the North Pacific, is not in favor of the eastward propagation of Rossby waves from the North Pacific into the Eurasian continent (Supplementary Fig. 9a, b). Previous studies have reported that these tropospheric circulation changes may further inhibit planetary wave upward propagation into the stratosphere, in turn strengthening the SAV^23,26^.

To further confirm the influence of the warmer CNP SST on the SAV strength, we conducted two numerical simulations using the Whole Atmosphere Community Climate Model, version 4 (WACCM4), which is the atmospheric component of the Community Earth System Model (CESM). Two 50-year time-slice simulations (a reference run and a sensitivity run), which only differ in their CNP SST, are designed. More details about the numerical model and two numerical experiments can be found in the Methods section.

**Fig. 5:**
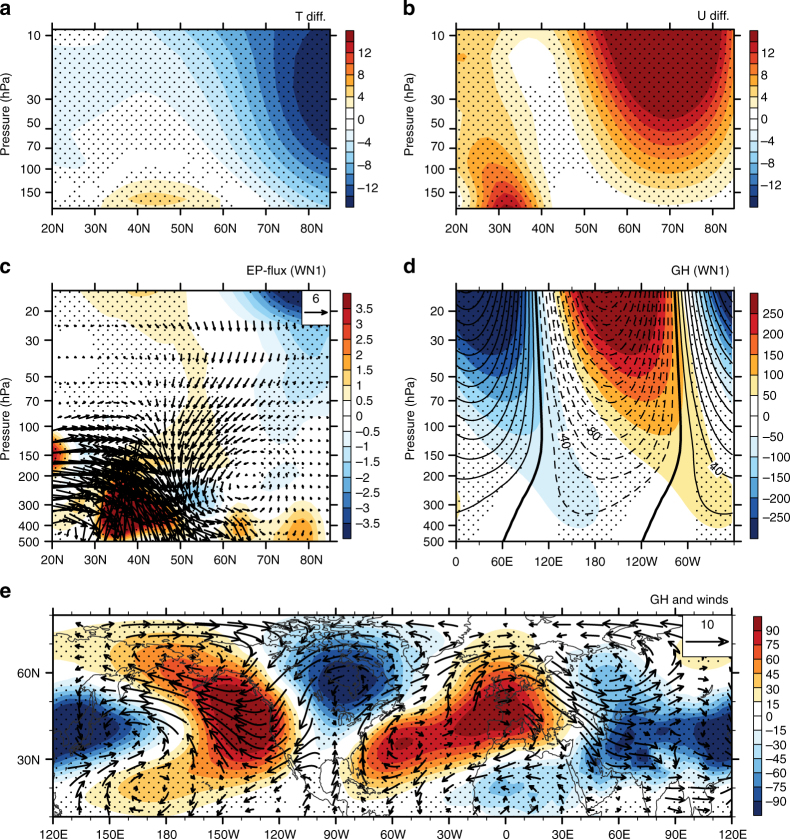
Model responses to the warmer central North Pacific SST. Differences in the physical quantities between the sensitivity and reference runs: **a** temperature differences; **b** zonal wind differences; **c** Eliassen–Palm (EP) flux vectors (arrows; units: 0.5×10^4^ kg s^−2^ for vertical vectors and 0.5×10^6^ kg s^−2^ for horizontal vectors) and their divergences (shading) for the wavenumber-1 component; **d** geopotential height departures averaged over 45°N–75°N for the wavenumber-1 component (contours); and **e** geopotential height (shading) and winds (vectors) at 500 hPa during boreal winter. Shaded contours in **d** show the mean climatology of the geopotential height of the wavenumber-1 component. The stippled areas indicate differences significant at/above the 98% confidence level

Circulation responses to the warmer CNP SST are presented in Fig. 5. As expected, a warmer CNP SST forces a stronger SAV. Relative to that in the reference run, temperature in the Arctic stratosphere decreases significantly (Fig. 5a), accompanied by stronger zonal winds (Fig. 5b) in the run, with a warmer CNP SST. As a result of the atmospheric response to the warmer CNP SST, the upward wave flux from the troposphere into the stratosphere associated with the wavenumber-1 planetary wave is depressed in the run, with a warmer CNP SST. This weakened-upward wave flux in the stratosphere is consistent with the out-of-phase variability of forced and climatological geopotential height anomalies associated with the wavenumber-1 planetary wave. That is, forced geopotential height anomalies in response to the CNP SST increase interfere destructively with climatological geopotential height anomalies associated with the wavenumber-1 planetary wave, and hence result in a weakened wavenumber-1 planetary wave in the troposphere and reduced wave fluxes in the stratosphere (Fig. 5d). The model results also show that the increase in CNP SST tends to weaken the Aleutian low, as is suggested by the increase in the geopotential height and the anomalous anticyclonic circulation at 500 hPa over the North Pacific in response to the increase in CNP SST (Fig. 5e).

To quantify the contribution of the warming trend of CNP SST to the strengthening trend in SAV during 1998–2016, we perform a multiple linear regression analysis on interannual timescales from observations and reanalysis (see Multiple linear regression analysis and Computation of the central North Pacific contribution in the section of Methods), and also conduct a numerical experiment to estimate the contributions of warming in the CNP to SAV strengthening (see Estimation of the central North Pacific contribution in the section of Methods). Results from observation and model simulation both indicate that the warming trend of CNP makes a contribution of ~25% to the strengthening trend in SAV during 1998–2016.

## Discussion

In this paper, we investigate the trends in the SAV during the past two decades by using three different reanalysis datasets and a chemistry-climate model. During 1997/98–2015/16, the SAV had a strengthening trend, which is different from the weakened SAV during 1979–2008^10^. The strengthening of the SAV is closely related to the warming of SSTs over the CNP, which acts to reduce the strength of the wavenumber-1 planetary wave in the troposphere and causes a decrease in the wave flux in the Arctic stratosphere. The warming of the CNP SST tends to weaken the Aleutian low, which interferes destructively with the climatological wavenumber-1 planetary wave and weakens the wavenumber-1 planetary wave in the troposphere. Previous studies have discussed the connections between the SAV and the North Pacific SST—in particular, the PDO^23,26,43,44^. However, the trend in the SAV during 1998–2016 has not been discussed to date and, hence, we focus on the recent-strengthening trend in the SAV. This recent SAV strengthening is partly attributable to the warming trend in CNP SST, which modifies the wavenumber-1 planetary wave. The contribution of the warming trend in the CNP SST to the strengthening trend in the SAV during 1998–2016 is ~25%.

The warming of the CNP SST may not be the only factor responsible for the strengthening of the SAV during the past decades, and other factors—including ENSO^24,25,33^, greenhouse gases^9^ such as stratospheric ozone^28^, sea ice^4,45,46^, Quasi-Biennial Oscillation^47^, solar activity^48^, and interhemispheric oscillations^49,50^—may also affect the variability of the SAV strength. During recent decades, accelerated near-surface warming of the northern high-latitude (Arctic amplification) has attracted extensive attention^38,45,46,51,52^. Studies have demonstrated that the sea-ice loss—particularly over the Barents and Kara seas (BKS)—will result in a weakened SAV, accompanied by a negative Arctic oscillation in the troposphere^8,46,53^. Recently, Nakamura et al.^46^ confirmed a crucial role played by the stratosphere in the impact of Arctic sea ice on the mid-latitude climate. However, other studies have reported that Arctic sea-ice loss will lead to a cooler and stronger SAV^54,55^. Another recent study pointed out that the decline in the sea ice over the BKS could enhance the moisture intrusions into the Arctic, further resulting in an increase in downward infrared radiation^52^, suggesting a cooling of the stratosphere. Apparently, the impacts of the sea-ice loss on the SAV strength is still under debate and the precise nature of the relationship between the SAV and the sea-ice loss, and even the Arctic amplification, are still not completely understood. Therefore, the systematic impacts on the SAV of all different factors mentioned above need to be examined further in the future work.

Recent studies have shown that a weakened SAV, which is often followed by a negative phase of the Arctic Oscillation at the surface^6^, leads to more frequent cold air outbreaks from high- to mid-latitudes^4,7,8^. Some previous studies have shown that the conditions in the lower stratosphere are important for downward coupling to the troposphere^56–58^. The composite differences in the surface temperature between the cold and warm SAV show a pattern with warm Arctic and cold continents (Supplementary Fig. 11). A cooling in the North America associated with the cold SAV is consistent with the Kosaka and Xie^59^, which showed a cooling trend in the North America in boreal winter during 2002–2012. Note that the northern Eurasian continent associated with the cold SAV gets warming, which is in accordance with the slight warmed trends in the north of the Eurasian continent during 1998–2016 in DJF (Supplementary Fig. 12). It suggests that the warming in the north of the Eurasian continent during 1998–2016 in DJF is possibly related to the strengthened SAV. But the exact relationships and mechanisms between the SAV and the surface climate in the mid-latitude continents deserves further investigations in the future.

Understanding the link between the tropospheric forcing and SAV is essential for understanding the coupling between the stratospheric and the tropospheric climate, and our comprehension of the stratosphere-troposphere coupling is likely to improve the winter-time forecasts^60,61^. Here our results suggest that the warming CNP SST has tended to strengthen the SAV during the past two decades, which may influence the temperature in the mid-latitude continents. If the CNP SST continues to warm in the future, the SAV may still strengthen further, followed by less cold air from high-latitudes into the mid-latitudes and lead to less frequently cold winters at mid-latitudes.

## Methods

### Trend analysis

As the SAV is most pronounced in DJF in the Northern Hemisphere, this study is restricted to boreal winter. Because non-parametric methods are less sensitive to outliers, the Sen median slope and the Mann–Kendall method are adopted to estimate the trends and their statistical significance, respectively.

### Model and simulations

The WACCM extends from the surface to approximately 150 km, and has a detailed middle-atmosphere chemistry^62^. The fifty-year time-slice simulations in the present work use a horizontal resolution of 1.9° × 2.5° lat. × lon. with chemistry processes switched off. In the reference experiment, we use the observed monthly mean climatological SST from the UK Met Office Hadley Centre based on the period 1950–2010. Monthly mean climatologies of surface emissions (including greenhouse gases and ozone-depletion substances) used in the reference experiment are obtained from the Inter-governmental Panel on Climate Change (IPCC) AR4 A1B emissions scenario. The configurations in the sensitivity run are the same as those in the reference run, except for the SST over the CNP (35°N–45°N, 150°E–150°W), where warmer SST anomalies (SSTAs) are superimposed. These superimposed SSTAs over the CNP are obtained as differences in SSTAs between stronger negative PDO phases (normalized PDO index ≤−0.8) and stronger positive PDO phases (normalized PDO index ≥0.8) during 1950–2014. To examine the effects of the SST over the CNP on the SAV strength in a more straightforward manner, and to exclude impacts of sea-ice changes, two numerical experiments use the same sea-ice field.

### Multiple linear regression analysis

To further clarify the roles of different factors in modulating the trends in the SAV, a multiple linear regression analysis is performed on the SAV time series for the period of 1998–2014. Let *X*_1_, *X*_2_, *X*_3_, *X*_4_, *X*_5_, *X*_6_, and *X*_7_ denote the standardized CNP SSTs (averaged over 35°N–45°N; 150°E–150°W, from which the ENSO signals have been removed using the linear regression method), Nino3.4 index, QBO index at 30 hPa, CO_2_, sea-ice concentration (SIC; averaged over the north of 65°N), stratospheric aerosol (532 nm, aerosol optical depth, AOD; averaged over 65°N–90°N and 50–10 hPa), and solar cycle (SC; represented by the 10.7 cm solar flux) index, respectively. Then the multiple linear regression is expressed as:

1 $${\mathrm {SAV}}(\lambda ,\varphi ,t) = \mathop {\sum}\limits_{{{i = 1}}}^{\mathrm{7}} {\alpha _{i}} X_i(t) + {\mathrm{residual}}$$

It is found that the ratio of the total variance of $\mathop {\sum}\nolimits_{i = 1}^{i = 7} {\alpha _i} X_i(t)$ to the variance of the SAV averaged over Arctic region (65°N–90°N) is 54.79%, indicating that more than half of the variance of the SAV can be explained by the seven factors, as in total. Note that these seven factors are almost independent of each other because the correlation coefficients between each pair of the seven factors during 1998–2014 (Supplementary Table 1) are statistically insignificant. Different factors have different impacts on SAV variations, among which the impacts of CNP SSTs are the largest. This can be seen in the regression patterns for $\alpha _{{i}}(\lambda ,\varphi )\,(i = 1,2,3......7)$ in Eq. (1), as obtained by performing multiple linear regressions (Supplementary Fig. 13). The regressed trends in the Arctic stratospheric temperature well captures the major features of stratospheric Arctic temperature trends during 1998–2014, exhibited in the NCEP2 (Supplementary Fig. 13a); the pattern correlation between Supplementary Fig. 13a and Supplementary Fig. 13b reaches 0.97. Meanwhile, the contribution of the CNP SSTs to the SAV trend is the largest among the seven factors examined here.

To quantify the relative contributions of the above mentioned factors to the SAV trend, according to Lin et al.^63^, a fraction is defined as area-weighted ratio of regressed trends for different factors to the area-weighted SAV trends over the region (65°N–90°N), which is written as:

2 $$f_i = \mathop {{\int\int}}\limits_\Sigma {{\mathrm {Trend}}_{X_{i}}\alpha _i(\lambda ,\varphi ){\mathrm{cos}}\varphi {\mathrm{d}}\varphi {\mathrm{d}}\lambda } /\mathop {{\int\int}}\limits_\Sigma {{\mathrm{Trend}}_{\mathrm {sav}}{\mathrm {cos}}\varphi {\mathrm{d}}\varphi {\mathrm{d}}\lambda }$$

where ${\mathrm{Trend}}_{X_{{i}}}$ is the trend of *X*_1_, *X*_2_, *X*_3_, *X*_4_, *X*_5_, *X*_6_, and *X*_7_ indices, respectively, ${\mathrm{Trend}}_{\mathrm{sav}}$ represents the trend of the temperature in the Arctic stratosphere. According to Eq. (2), the area-weighted relative contributions of each index to the stratospheric Arctic temperature trends are 25.4% (CNP SSTs), 5.2% (CO_2_), 3.9% (QBO), –4.2% (SIC), –0.5% (ENSO), –0.5% (SC), 0.02% (AOD), respectively. In this way, the total trend contribution from the seven factors to the observed SAV trends is found to be 54.79% over Arctic region. From the above analysis, we can conclude that the potential contribution of the CNP SST changes on SAV trend is about 25%.

### Computation of the central North Pacific contribution

We also tried to quantify the contribution of CNP SST changes to the SAV variations on interannual timescales. As the CNP SSTs, QBO, and SIC indices during 1998–2014 exhibit significant interannual variations and apparent trends (figure not shown) and have evident contributions to the SAV trend (Supplementary Fig. 13), the interannual variations of the stratospheric Arctic temperature, CNP SSTs, SIC, and QBO indices are shown in Supplementary Fig. 14. Here, a 7-year high-pass filter is applied to the stratospheric Arctic temperature, CNP SSTs, SIC, and QBO to remove signals on time scales longer than 7 years. The fluctuations of the stratospheric Arctic temperature (Supplementary Fig. 14a) appear to be out-of-phase with those in the CNP SSTs (Supplementary Fig. 14b) during the period from 1979 to 2014 on interannual timescales. The correlations of the stratospheric Arctic temperature averaged over 65°N–90°N and 50–10 hPa respectively with CNP SSTs, SIC, CO_2_, and QBO indices during two periods, 1979–2014 and 1998–2014 on interannual timescales are shown in Supplementary Table 2. High correlation coefficients between the stratospheric Arctic temperature and CNP SSTs are observed with an anticorrelation coefficient of –0.50 during 1998–2014, suggesting that the variations of CNP SSTs make a significant contribution to the stratospheric Arctic temperature changes by about 25% during 1998–2014 on interannual timescales.

### Estimation of the central North Pacific contribution

To estimate the contribution of the CNP SSTs to the SAV strengthening, we also designed another experiment (named CNP_obs), which adopts the observed SSTs over the CNP. The trends in the geopotential height and wind anomalies averaged over 50–10 hPa over the Arctic in CNP_obs in DJF during 1979–2005 exhibit a wavenumer-1 like pattern (Supplementary Fig. 15), which indicate that a warming trend in the CNP SSTs could lead to a strengthening trend in the SAV. The trend in the observed SSTs over the CNP during 1979–2005 is 0.19 K decade^–1^ and the trend in the geopotential height averaged over 65°N–90°N and 50–10 hPa (PCH) in CNP_obs during 1979–2005 is –11.56 gpm decade^–1^. From a linear point of views, if a warming trend in the CNP was 1.0 K decade^–1^, it would result in a decreasing trend of –60.84 gpm decade^–1^ in PCH. The trend of observed CNP SSTs during 1998–2016 is 0.58 K decade^–1^, which would lead to a decreasing trend in PCH by (−60.84 gpm·K^−1^) × 0.58 K·decade^−1 ^≈ −35.29 gpm·decade^−1^. The trend of PCH from MERRA during 1998–2016 is −142.08 gpm decade^−1^, which implies that (−35.29 gpm·decade^−1^) ÷ (−142.08 gpm·decade^−1^) × 100% ≈ 25% of the observed trend in the PCH is contributed from the warming in the CNP.

### Code availability

All the plots in this study are made using software National Center for Atmospheric Research (NCAR) Command Language (NCL; available at http://www.ncl.ucar.edu/), which is a free software developed by the NCAR.

### Data availability

All observational data used in this article are publicly available and the data supporting the findings of this study are available upon request. We use the monthly mean temperature, winds, velocity wind velocity, and geopotential height from (1) the MERRA reanalysis dataset for 1998–2016 with a horizontal resolution of 1.25° × 1.25° lon. × lat. (available online at http://disc.sci.gsfc.nasa.gov/daac-bin/FTPSubset.pl), (2) the ERA-Interim dataset for 1998–2016 with a horizontal resolution of 1.5° × 1.5° lon. × lat. (http://apps.ecmwf.int/datasets/data/interim-full-moda/levtype=pl/), (3) the NCEP2 dataset for 1998–2016, which has a horizontal resolution of 2.5° × 2.5° lon. × lat. (https://www.esrl.noaa.gov/psd/data/gridded/data.ncep.reanalysis2.pressure.html). We also used the SST data from the ERSST.V3b, which has a 2° × 2° lon. × lat. grid mesh and covers the period from 1998 to 2016 (http://www.metoffice.gov.uk/hadobs/hadisst/data/download.html) and the PDO index (http://research.jisao.washington.edu/pdo/PDO.latest.txt). There are some indices used in the Supplementary Information: (i) the Niño-3.4 index (https://www.esrl.noaa.gov/psd/data/correlation/nina34.data), (ii) the QBO index (https://www.esrl.noaa.gov/psd/data/correlation/qbo.data), (iii) the sea ice concentration (https://www.metoffice.gov.uk/hadobs/hadisst/data/download.html), (iv) the CO_2_ concentration adopted from the IPCC AR4 A1b scenarios^64^, (v) the stratospheric aerosols (ftp://iacftp.ethz.ch/pub_read/luo/ccmi/WACCM_sad/), (vi) the solar flux (https://www.esrl.noaa.gov/psd/data/correlation/solar.data), (vii) the surface temperature from CRU (https://www.esrl.noaa.gov/psd/data/gridded/data.crutem4.html) and GISS (https://www.esrl.noaa.gov/psd/data/gridded/data.gistemp.html).

## Electronic supplementary material

Supplementary Information
