# Supplementary material for: Recent strengthening of the stratospheric Arctic vortex response to warming in the central North Pacific
Source: Nat Commun. 2018 Apr 27;9:1697. doi: 10.1038/s41467-018-04138-3 (PMC5923267; doi:10.1038/s41467-018-04138-3)
Supplement: Supplementary file 1 — Supplementary Information [file 41467_2018_4138_MOESM1_ESM.pdf]

Supplementary Information for

Recent strengthening of the stratospheric Arctic vortex response to warming in the central North Pacific

Hu et al.

This file includes:

Supplementary Figures 1–15 and Tables 1–2.

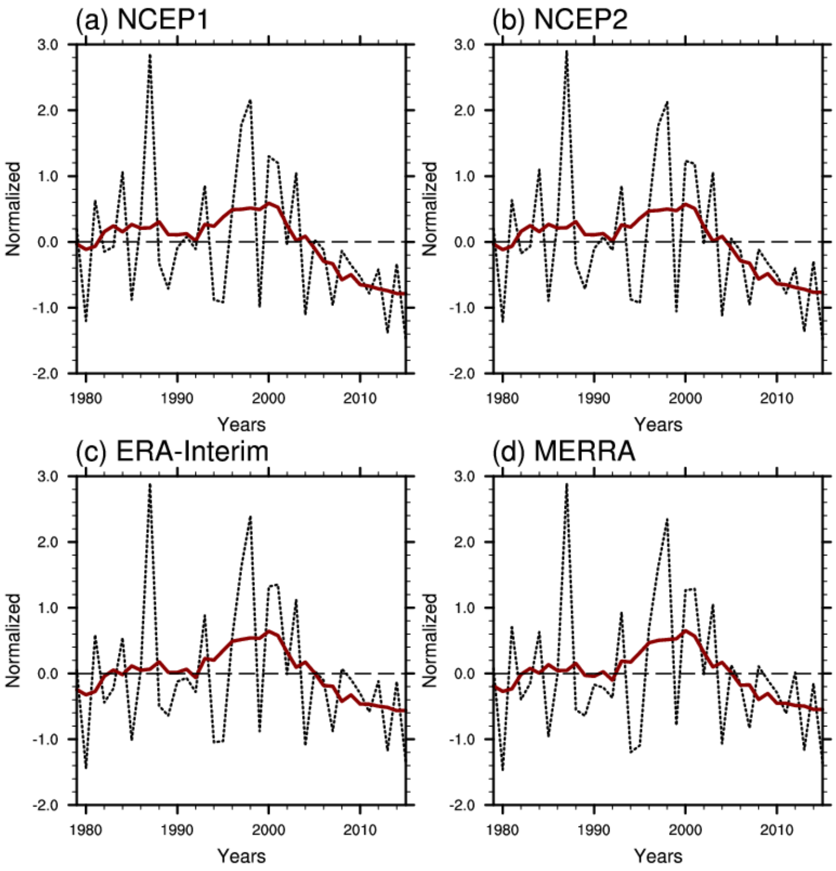

Supplementary Figure 1. The normalized time series of the stratospheric temperature averaged from 50 hPa up to 10 hPa over 65°N–90°N during the period 1979–2015 in December derived from (a) NCEP1, (b) NCEP2, (c) ERA-Interim, and (d) MERRA. The red lines are for the 10-year running mean of the normalized polar cap temperature in the Arctic stratosphere.

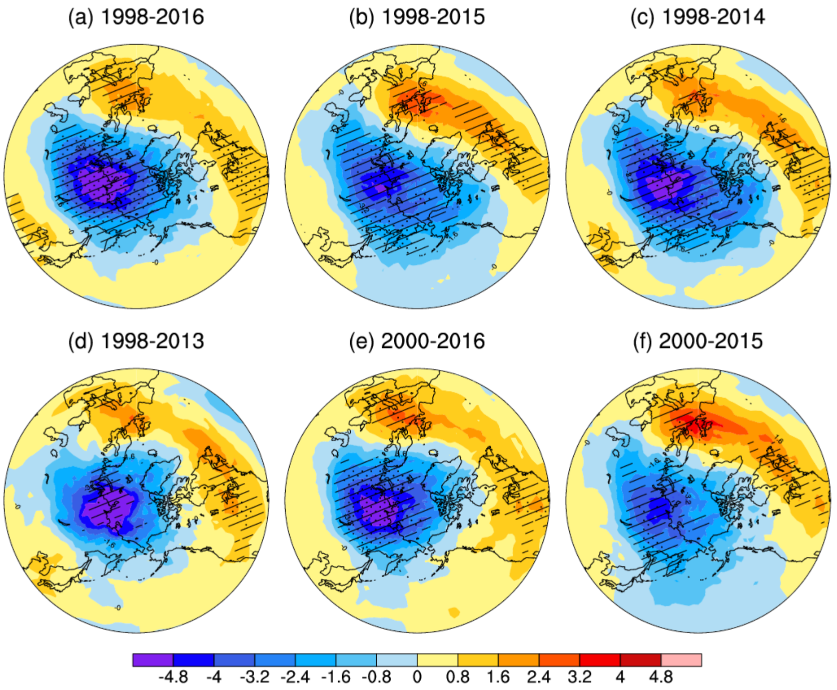

Supplementary Figure 2. Trends (units: K decadal^{-1}) in the stratospheric Arctic temperature averaged over 50–10 hPa from NCEP2 in DJF during the periods (a) 1998–2016, (b) 1998–2015, (c) 1998–2014, (d) 1998–2013, (e) 2000–2016, and (f) 2000–2015. Hatched (stippled) areas are for values at/above 90% (95%) level of confidence.

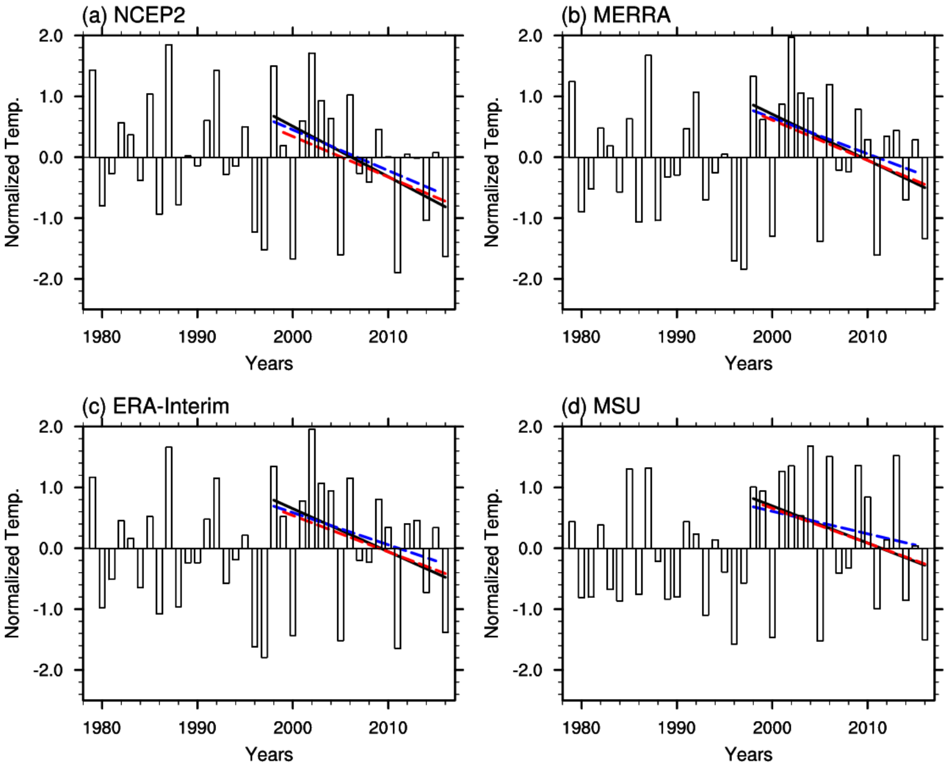

Supplementary Figure 3. The normalized time series of stratospheric temperature averaged over 65°N–90°N and 50–10 hPa using datasets including (a) NCEP2, (b) MERRA, (c) ERA-Interim, and (d) MSU RSS T4. The straight black, red, and blue lines are for the linear trends in the normalized stratospheric Arctic temperature during 1998–2016, 1999–2016, and 1998–2015, respectively.

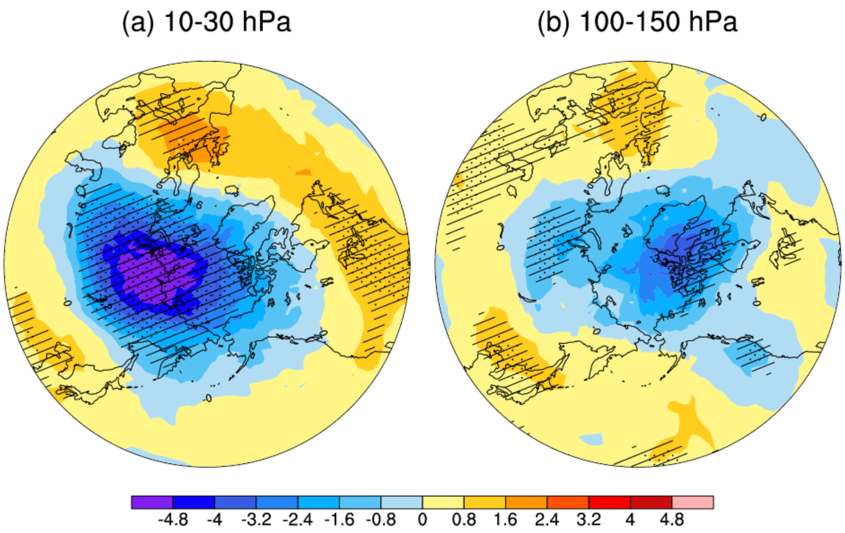

Supplementary Figure 4. Trends (units: K decadal^{-1}) in the temperature averaged over (a) 10–30 hPa and (b) 100–150 hPa from NCEP2 in DJF during 1998–2016. Hatched (stippled) areas are for values at/above 90% (95%) level of confidence.

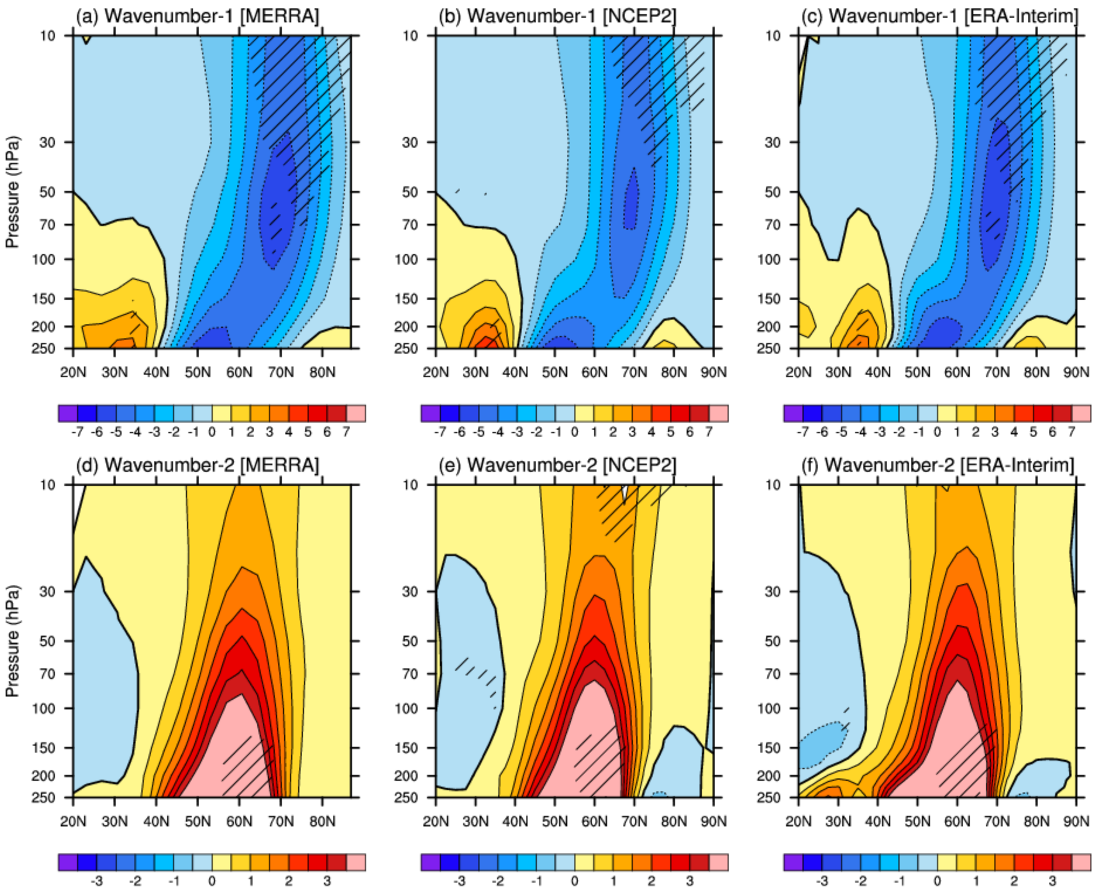

Supplementary Figure 5. The vertical component of EP flux in unit of 10^{-3} kg s^{-2} decade^{-1} for trends of the wavenumber-1 (a–c) and wavenumber-2 (d–f) components in DJF during the period 1997/98–2015/16 as derived from MERRA (left panels), NCEP2 (middle panels), and ERA-Interim (right panels) reanalysis datasets. Hatched areas are for the trends significant at the 95% level.

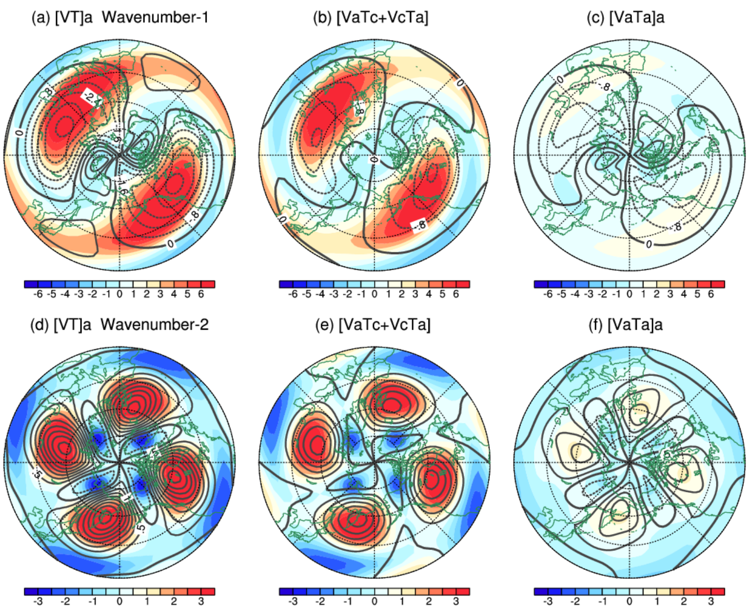

Supplementary Figure 6. Trends (shading, units: K m s^{-1} decadal^{-1}) and climatological mean (line contours, units: K m s^{-1}) in the (a–c) wavenumer-1 and (d–f) wavenumber-2 components of (a, d) eddy heat flux $(V^{*}T^{*})_{a}$ (left panels), (b, e) interference term $(V_{a}^{*}T_{c}^{*}+V_{c}^{*}T_{a}^{*})$ (middle panels), and (c, f) wave-packet term $(V_{a}^{*}T_{a}^{*})_{a}$ (right panels) at 200 hPa in DJF during the period 1997/98–2015/16. The contour intervals of climatological mean in the wavenumber-1 and wavenumber-2 components are 0.8 K m s^{-1} and 0.5 K m s^{-1}, respectively. Here, the subscripts c and a denote the climatological mean and anomalies, respectively. The asterisks are defined as deviations from their zonal means. The interference term $(V_{a}^{*}T_{c}^{*}+V_{c}^{*}T_{a}^{*})$ represents contributions from the interference between the climatological mean planetary waves and anomalies and the wave packet term $(V_{a}^{*}T_{a}^{*})_{a}$ represents an anomalous instantaneous contribution from the anomalous wave packet propagation.

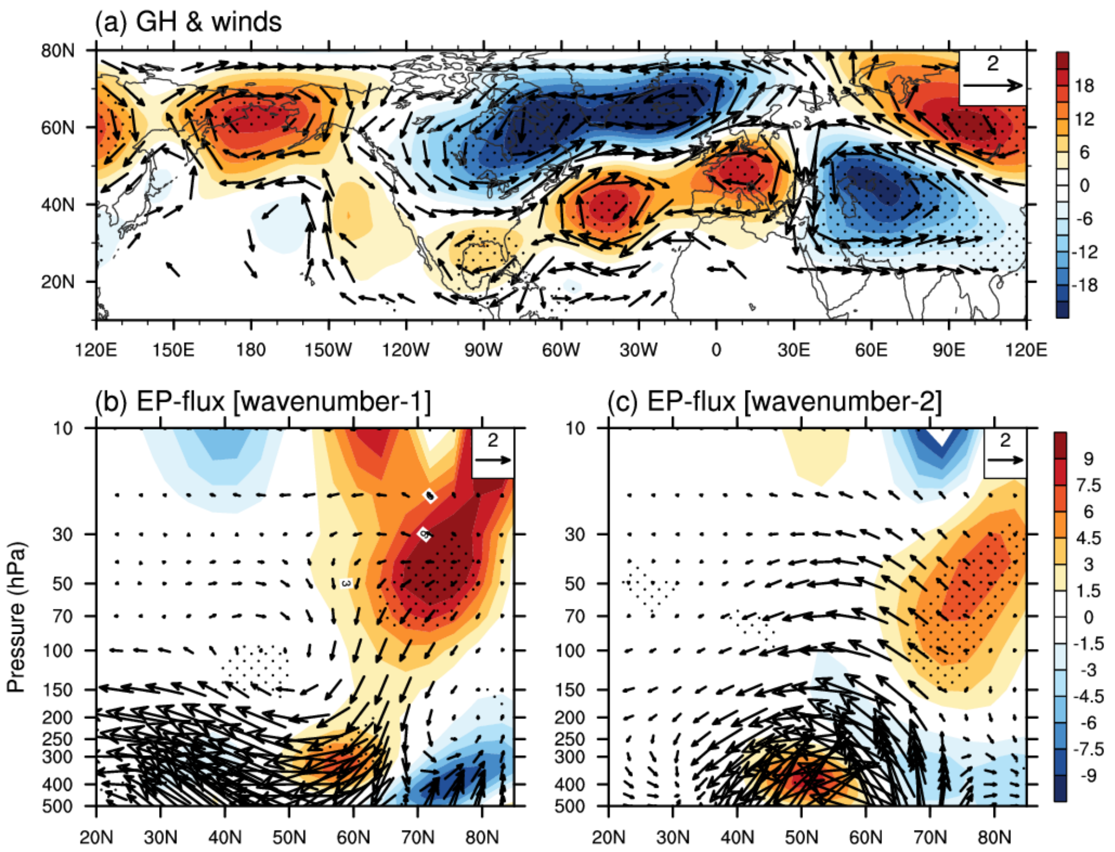

Supplementary Figure 7. The regressed geopotential height in 100 gpm (shaded contours) and horizontal wind vectors (larger than 0.5 m s^{-1} shown) as obtained by regressing these quantities upon the normalized NPCH index in DJF during 1997/98–2015/16 (a). Shown in (b–c) are the same as in (a) but for the EP flux and its divergence for the regressed waves with wavenumber-1 (b) and with wavenumber-2 (c) components, respectively. The values in the stippled areas are significant at/above the 90% confidence level. The meaning of NPCH index is interpreted in the text of the article.

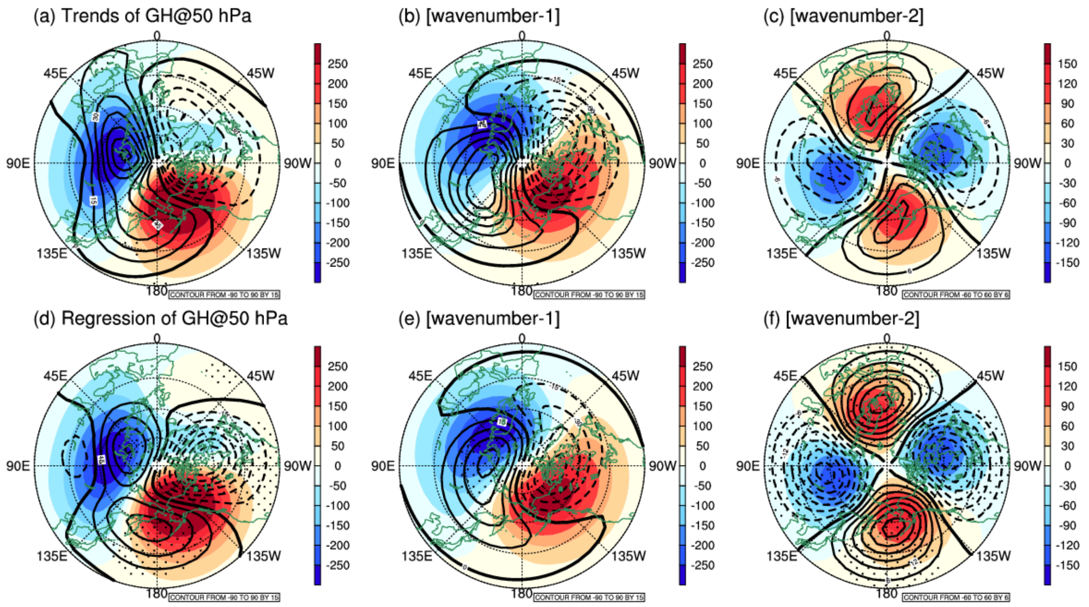

Supplementary Figure 8. Trends (contours) in the 50 hPa geopotential height for the zonal deviations (a), wavenumber-1 component (b), and wavnumber-2 component (c) in DJF derived from MERRA during the period 1997/98–2015/16. Shown in (d–f) are the same as in (a–c) but for the regression of zonal deviations (d), wavenumber-1 component (e), and wavnumber-2 component (f) on the normalized time-series of SST_{CNP}. Shadings are for the climatological distributions of the zonal deviations (left panels), wavenumber-1 component (middle panels), and wavenumber-2 component (right panels) of the 50 hPa geopotential height. Stippled areas are for values significant at/above the 90% confidence level.

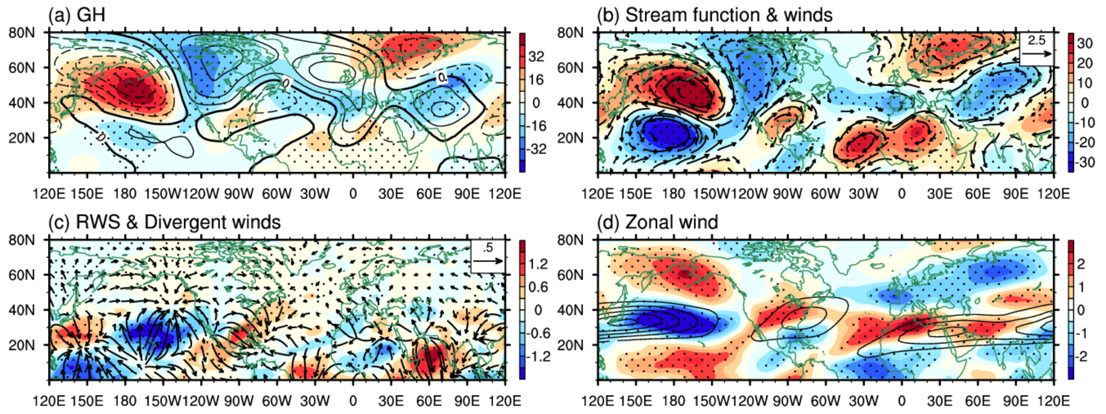

Supplementary Figure 9. Regression map of the detrended and 5-yr running averaged (a) geopotential height (shading), (b) streamfunction (shading; units: 10^5 m^2 s^{-1}) and winds (vectors; only the vectors above 0.5 m s^{-1} are shown), (c) Rossby wave source (shading, units: 10^{-11} s^{-2}) and divergent winds (vectors), and (d) zonal winds (shading, units: m s^{-1}) at 200 hPa from NCEP1 onto the normalized CNP SST in DJF during 1950–2014. The line contours in (a) represent the DJF climatological mean of the detrended and 5-yr running averaged geopotential height. The line contours in (d) represent the climatological mean of the 200 hPa zonal wind (only the values at and above 30 m s^{-1} are shown). The values over the stippled areas are statistically significant above the 95% level of confidence.

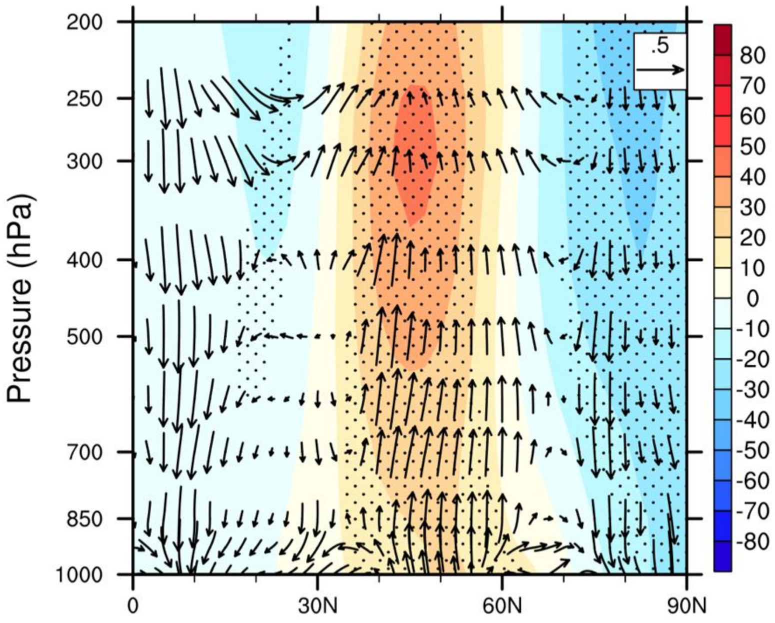

Supplementary Figure 10. Regression map of the detrended and 5-yr running averaged geopotential height (shading), and the vertical circulation (vectors) averaged over 150°E–150°W from NCEP1 onto the normalized CNP SST in DJF during 1950–2014. The vertical circulation is composed of the divergent meridional wind (units: m s^{-1}) and vertical velocity (units: mm s^{-1}). The stippled areas are for geopotential height anomalies significant at/above the 90% confidence level.

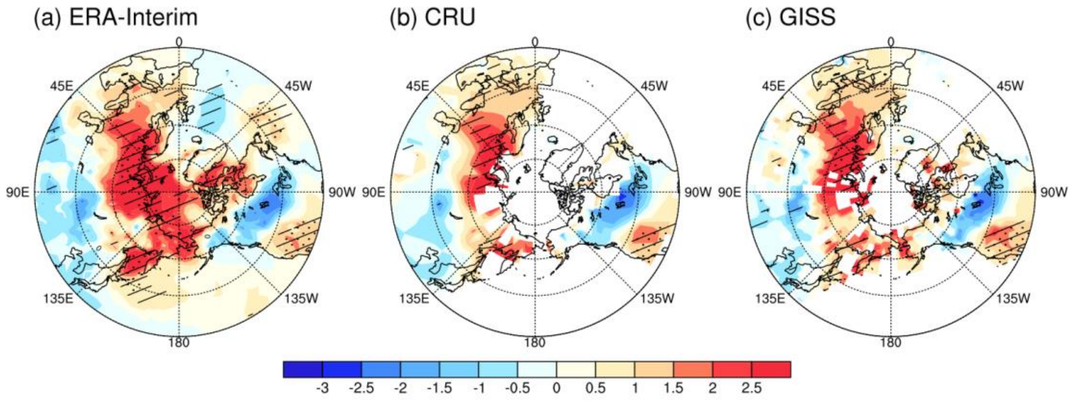

Supplementary Figure 11. Composite differences in surface temperature derived from ERA-Interim (a), CRU (b), and GISS (c) in DJF between cold and warm SAV. The cold (warm) SAV is identified if the normalized polar cap (averaged over 65°N–90°N) temperature anomalies averaged over 10–30 hPa are greater than one negative (positive) standard deviation of the polar cap temperature anomalies. Hatched (stippled) areas are for values at/above 90% (95%) level of confidence.

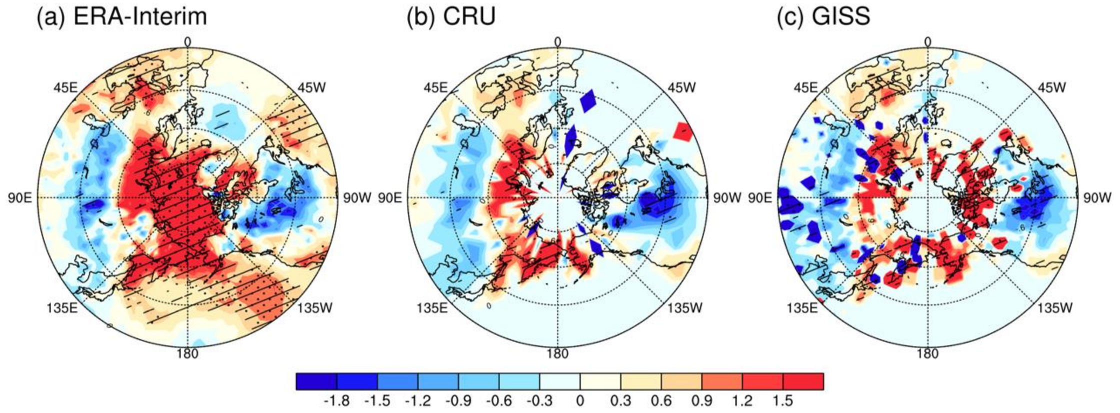

Supplementary Figure 12. Trends in the surface temperature derived from ERA-Interim (a), CRU (b), and GISS (c) during 1998–2016 in DJF. Stippled areas are for values at/above 90% level of confidence.

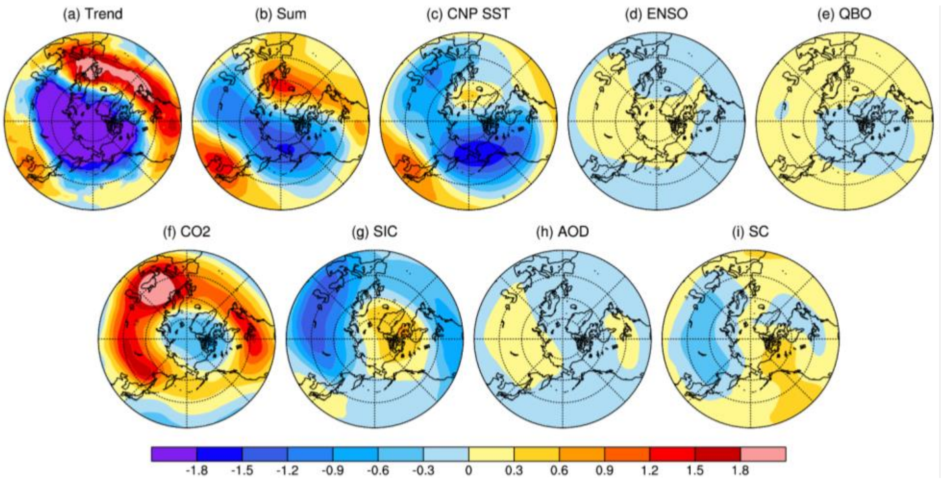

Supplementary Figure 13. (a) Trend pattern of the stratospheric Arctic temperature during 1998–2014 in DJF derived from NCEP2 (units: 0.5 K decadal^{-1}). (b) The trend pattern of the regressed temperature in the Arctic stratosphere. The trend patterns of the stratospheric Arctic temperature regressed separately on (c) CNP SSTs, (d) ENSO, (e) QBO, (f) CO2, (g) SIC, (h) SAD, and (i) SC during the period 1998–2014 in DJF.

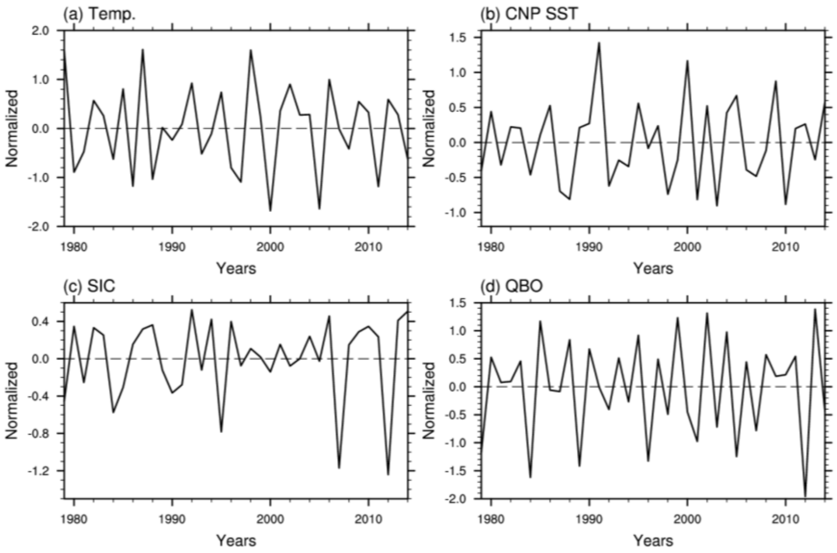

Supplementary Figure 14. The normalized time series of the inter-annual components of (a) stratospheric Arctic temperature, (b) CNP SSTs, (c) SIC, and (d) QBO indices during the period 1979–2014 in DJF.

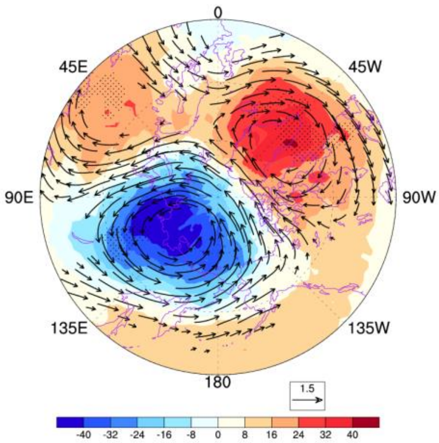

Supplementary Figure 15. Trends in the geopotential height (shading) and winds (vectors; only the values above 0.5 m s^{-1} $decade^{-1}$ are shown) averaged over 50–10 hPa in CNP_obs in DJF during the period 1979–2005. The values of geopotential height in the dotted areas are significant above the 90% confidence level.

Supplementary Table 1. Correlation coefficients between each pair of the indices including CNP SSTs, ENSO, QBO, CO_2, SIC, AOD, and SC during the period 1998–2014 on inter-annual timescales. Critical value at 95% confidence level is found to be 0.48 using a t-test.

Correlation coefficient	CNP SSTs	ENSO	QBO	CO_2	SIC	AOD	SC
CNP SSTs	1	–0.10	0.03	0.19	–0.05	–0.10	0.30
ENSO		1	–0.10	0.15	0.03	0.09	–0.18
QBO			1	–0.03	–0.18	0.04	0.18
CO_2				1	0.16	0.03	0.26
SIC					1	–0.10	–0.02
AOD						1	–0.25
SC							1

Supplementary Table 2. Correlations of the stratospheric Arctic temperature averaged over 65°N–90°N and 50–10 hPa with CNP SSTs, SIC, CO_2, and QBO indices, respectively for two periods, 1979–2014 and 1998–2014 on inter-annual timescales. Values with asterisks are for those at/above 95% confidence level.

Periods	CNP SSTs	SIC	QBO	CO_2
1979–2014	–0.34*	–0.07	0.06	–0.16
1998–2014	–0.50*	–0.01	0.18	–0.12
